# Supplementary material for: Reactivation of the PI3K/mTOR Signaling Pathway Confers Resistance to the FGFR4 Inhibitor FGF401
Source: Int J Mol Sci. 2025 Oct 9;26(19):9818. doi: 10.3390/ijms26199818 (PMC12525526; doi:10.3390/ijms26199818)
Supplement: Supplementary file 1 [file ijms-26-09818-s001.zip › ijms-3902931-supplementary.pdf]

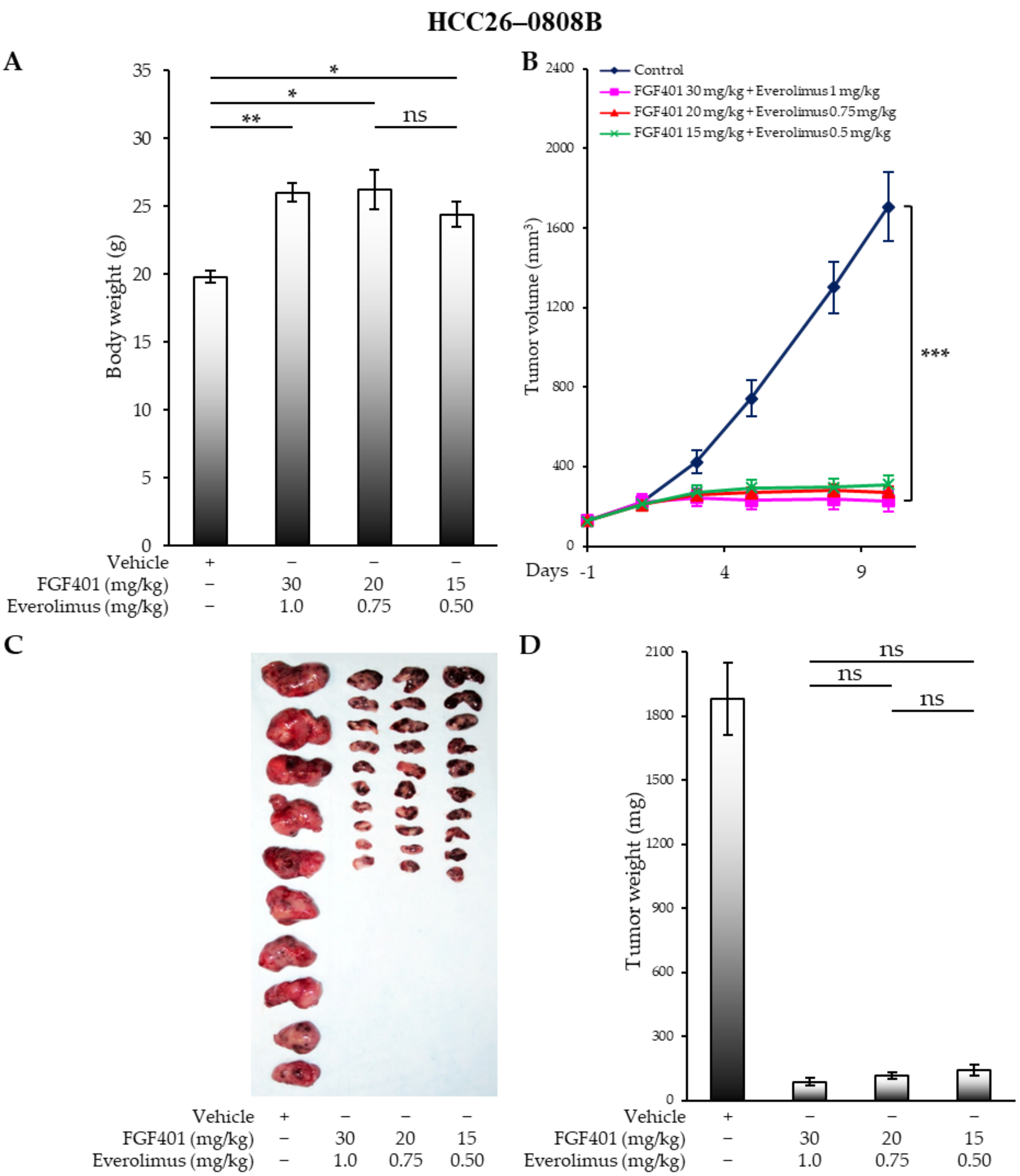

Supplementary Materials Figure S1. Dose-dependent effects of FGF401/everolimus on tumor growth in the HCC26–0808B model**.** Mice bearing HCC26–0808B xenografts (n = 8/group) were treated orally with vehicle or three step-down FGF401/everolimus combinations (30:1.0, 20:0.75, and 15:0.5 mg/kg) for 12 days. FGF401 was administered twice daily, whereas everolimus was administered once daily. A low-dose schedule of 30 mg/kg FGF401 was selected based on a previous study [19]. Treatment began when tumors reached ~100–250 mm^3. Tumor growth was monitored and tumor volume was calculated as described previously [74,75]. (**A**) Mean body weight ± SE at sacrifice, (**B**) mean tumor volume ± SE at indicated time points, (**C**) representative tumors from vehicle- and combination-treated groups, and (**D**) mean tumor weight ± SE are shown. Statistical significance was determined by one-way analysis of variance (ANOVA) followed by Tukey's test (* $p < 0.05$; ** $p < 0.01$; *** $p < 0.001$; ns, no significance).

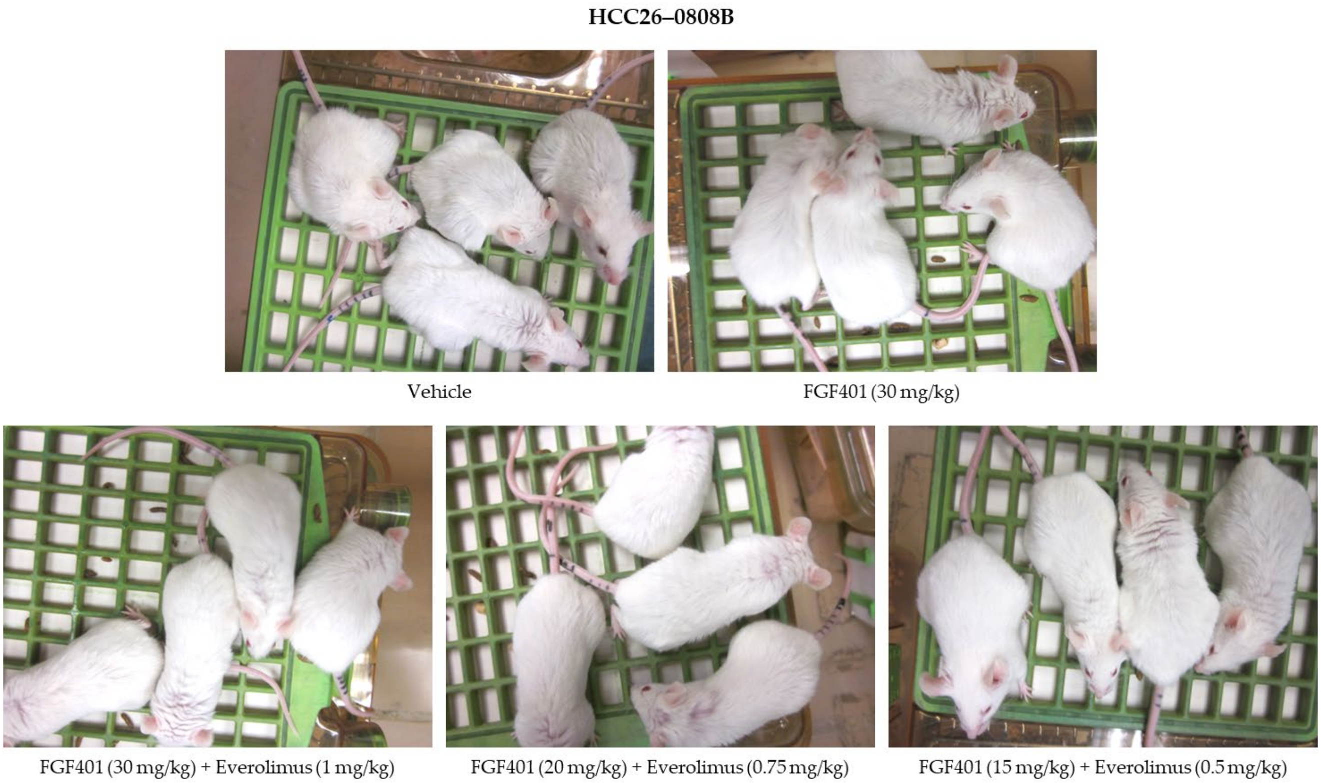

Supplementary Materials Figure S2. Effects of FGF401/everolimus on toxicity and side effects in mice**.** Mice bearing high-FGF19-expression HCC26–0808B xenografts (*n* = 8 mice/group) were treated orally with the vehicle, 30 mg/kg FGF401, or three step-down FGF401/everolimus combinations (30:1.0, 20:0.75, and 15:0.5 mg/kg) for 12 days as described in Supplementary Materials Figure S1. Across all treatment groups, mice exhibited a healthy coat, normal food and water intake, and normal social interactions and activity levels, and there were no signs of aggression among cage mates. These observations indicate that the administered dosages were well tolerated, with minimal toxicity and side effects.

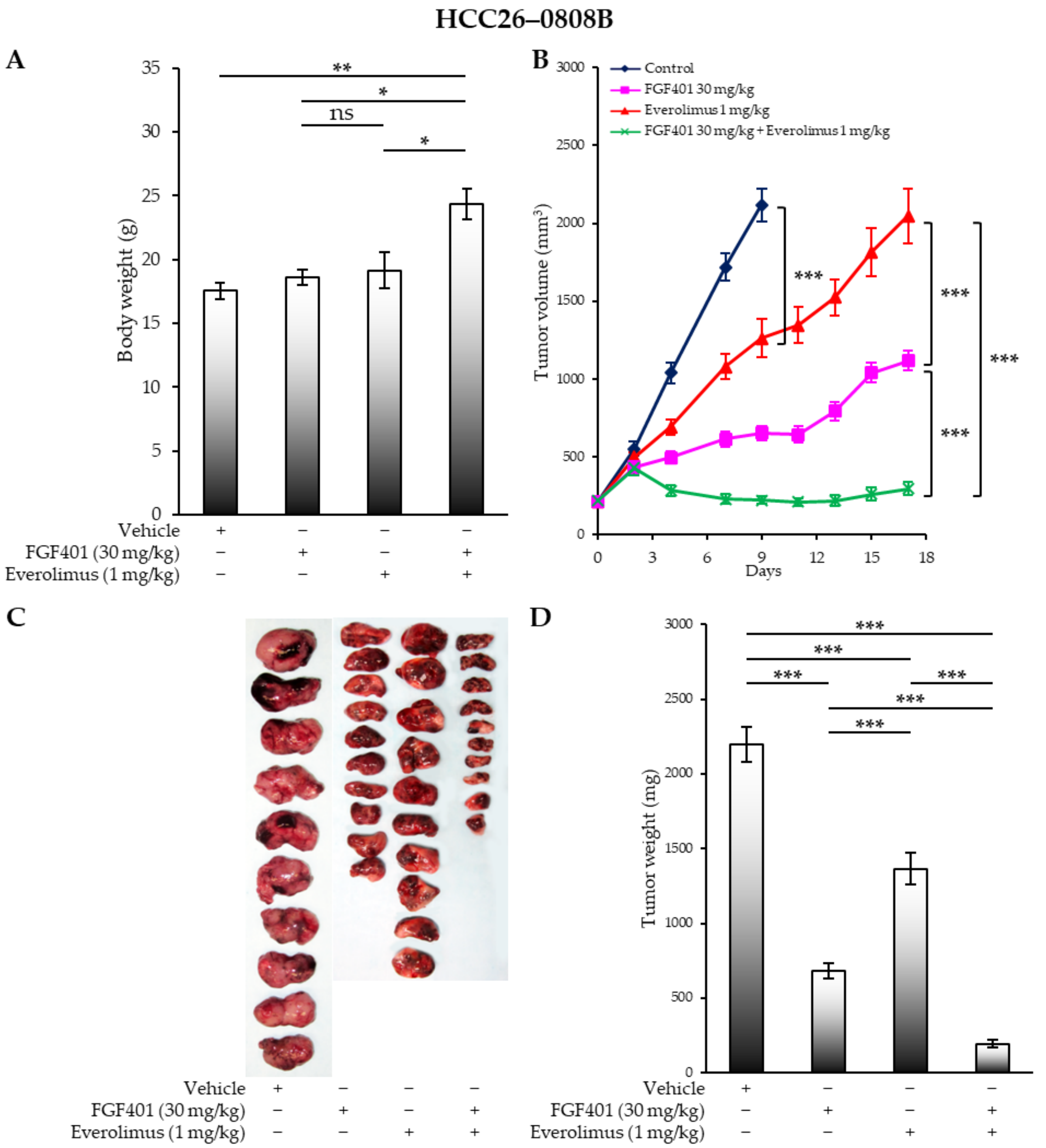

Supplementary Materials Figure S3. Effects of FGF401/everolimus on tumor growth in the HCC26–0808B PDX model. Mice bearing HCC26–0808B xenografts (*n* = 8–10/group) were treated orally with (a) vehicle (200 µl), (b) FGF401 (30 mg/kg, twice daily), (c) everolimus (1 mg/kg; once daily), or (d) FGF401 (30 mg/kg, twice daily) plus everolimus (1 mg/kg; once daily) for 18 days. Treatment was initiated when tumors reached ~100-250 mm^3. Tumor growth was monitored, and tumor volume was calculated as described previously [74,75]. (**A**) Mean body weight ± SE at sacrifice, (**B**) mean tumor volume ± SE at indicated time points, (**C**) representative tumors from each treatment group, and (**D**) mean tumor weight ± SE are shown. Statistical significance was determined by one-way analysis of variance (ANOVA) followed by Tukey's test (* $p < 0.05$; ** $p < 0.01$; *** $p < 0.001$; ns, no significance).

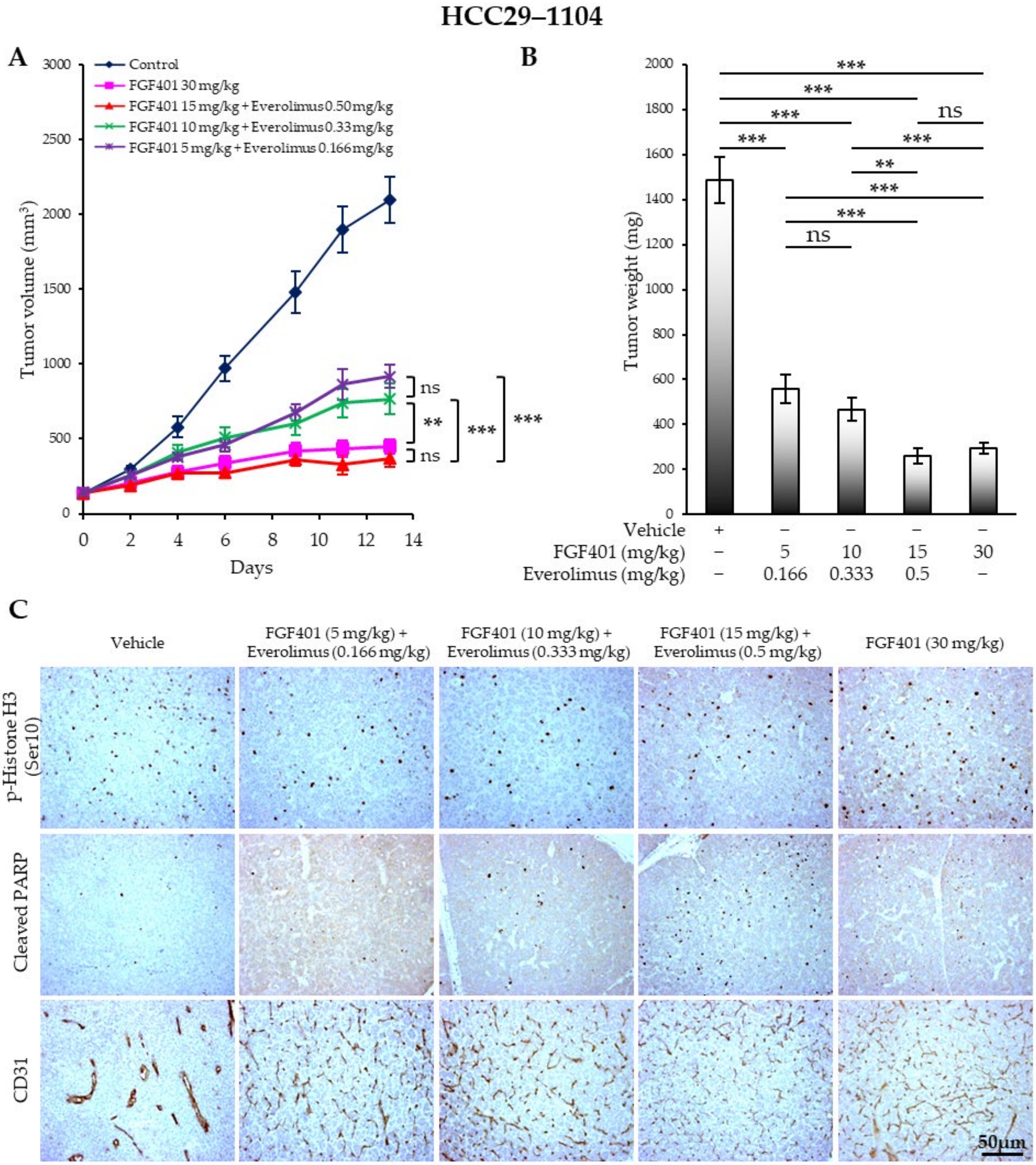

Supplementary Materials Figure S4. Effects of FGF401/everolimus on tumor growth, apoptosis, and blood vessel density in the HCC29–1104 PDX model. Mice bearing HCC29–1104 xenografts (n = 8/group) were treated orally with the vehicle (200 µl), FGF401 (30 mg/kg), or three step-down FGF401/everolimus combinations (15:0.5, 10:0.333, and 5:0.166 mg/kg) for 13 days. FGF401 was administered twice daily, whereas everolimus was administered once daily. Treatment was initiated when tumors reached ~100–250 mm3. Tumor growth was monitored, and tumor volume was calculated as described previously [74,75]. Tumor tissues collected 2 h after the last treatment were fixed in 10% buffered formalin, processed, and embedded in paraffin for IHC, as described in Section 4. Sections (5 µm) were immunostained with p-histone H3 (Ser 10), cleaved PARP, and CD31 antibodies to assess cell proliferation, apoptosis, and blood vessel density, respectively. (**A**) Mean tumor volume ± SE at indicated time points, (**B**) mean tumor weight ± SE are shown. Statistical significance was determined by one-way analysis of variance (ANOVA) followed by Tukey's test (** $p < 0.01$; *** $p < 0.001$; ns, no significance). (**C**) Representative tumor sections from vehicle- and drug-treated mice stained for p-histone H3 (Ser10), cleaved PARP, and CD31. Images were captured using an Olympus BX60 microscope (Olympus, Tokyo, Japan). Scale bars = 50 µm.

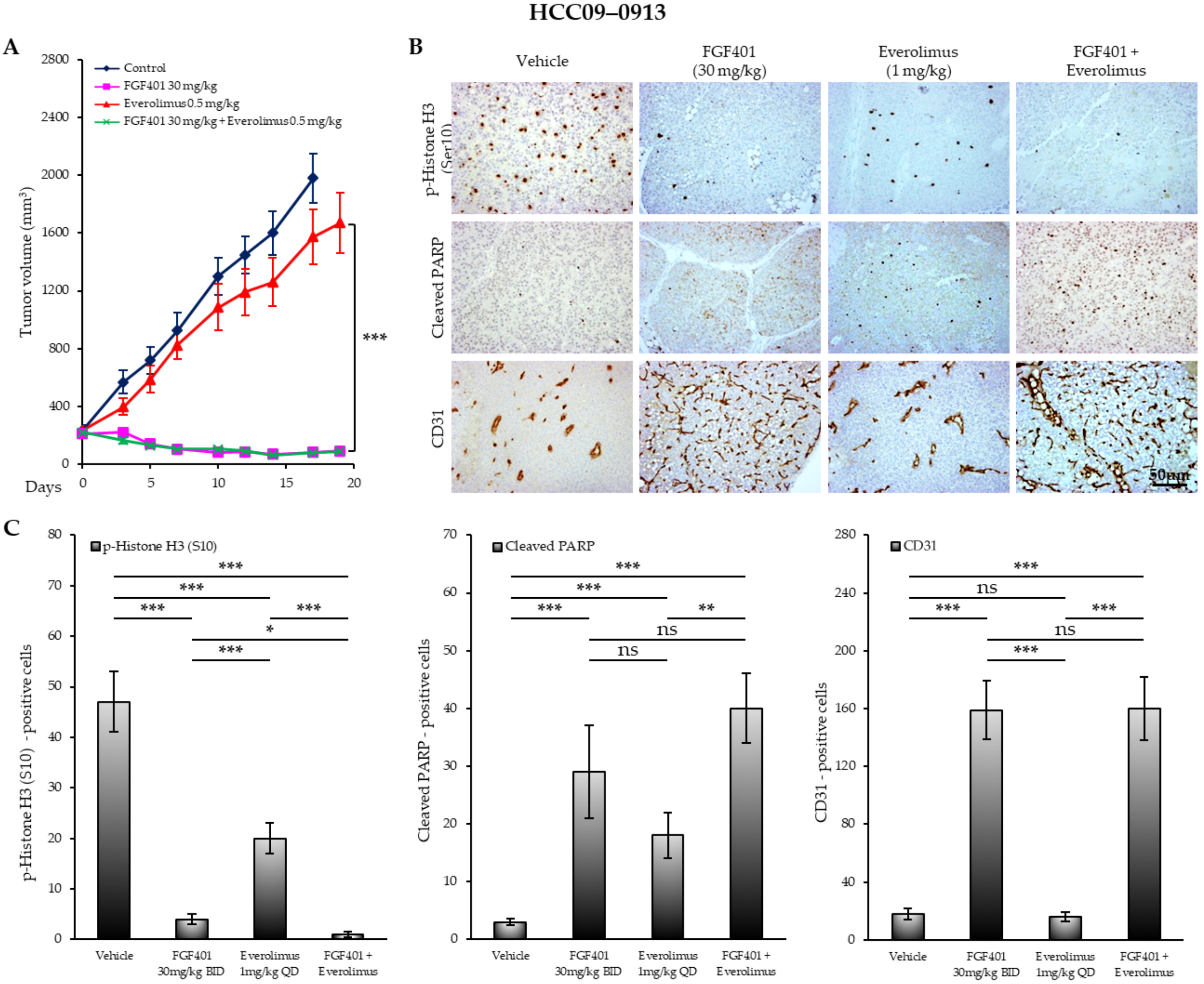

Supplementary Materials Figure S5. Effects of FGF401/everolimus on tumor growth, apoptosis, and total blood vessels in the HCC09–0913 PDX model. Mice bearing HCC09–0913 xenografts (*n* = 8/group) were treated orally with (a) vehicle (200 μl), (b) FGF401 (30 mg/kg, twice daily), (c) everolimus (1 mg/kg, once daily), or (d) FGF401 (30 mg/kg, twice daily) plus everolimus (1 mg/kg, once daily) for 18 days. Treatment was initiated when tumors reached ~100–250 mm3. Tumor growth was monitored, and tumor volume was calculated as described previously [74,75]. Tumor tissues collected 2 h after the last treatments were fixed in 10% buffered formalin, processed, and embedded in paraffin for IHC, as described in Section 4. Sections (5 μm) were immunostained with p-histone H3 (Ser 10), cleaved PARP, and CD31 antibodies to assess cell proliferation, apoptosis, and blood vessel density, respectively. (**A**) Mean tumor volume ± SE at indicated time points, (**B**) representative tumor sections from vehicle- and drug-treated mice stained for p-histone H3 (Ser10), cleaved PARP, and CD31 are shown. Images were captured using an Olympus BX60 microscope (Olympus, Tokyo, Japan). Scale bars = 50 μm. (**C**) Quantification of p-histone H3 (Ser10)-, cleaved PARP-, and CD31-positive cells. At least 10 randomly selected fields per slide were captured at 100× magnification using an Olympus BX60 microscope (Olympus, Tokyo, Japan). Positive cells were counted per field, and mean values ± SE are plotted. Statistical significance was determined by one-way analysis of variance (ANOVA) followed by Tukey's test (* $p < 0.05$; ** $p < 0.01$; *** $p < 0.001$; ns, no significance).

A

HCC26–1004

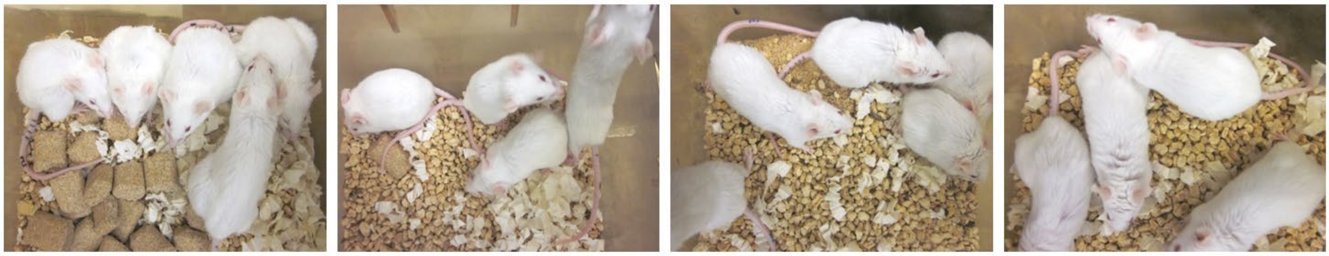

Vehicle | FGF401 (30 mg/kg) | Everolimus (1 mg/kg) | FGF401 + Everolimus

B

HCC26–0808B

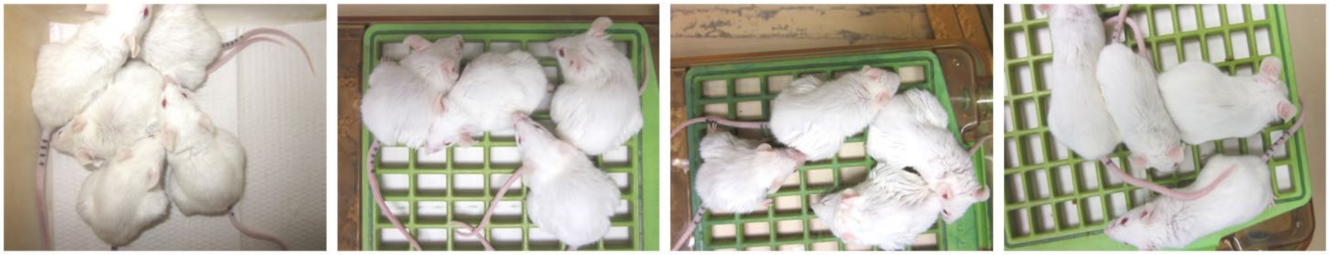

Vehicle | FGF401 (30 mg/kg) | Everolimus (1 mg/kg) | FGF401 + Everolimus

Supplementary Materials Figure S6. Toxicity and side effects of FGF401/everolimus in mice. Mice bearing HCC26–1004 and HCC26–0808B xenografts (*n* = 8/group) were treated orally with (a) vehicle (200 μl), (b) FGF401 (30 mg/kg, twice daily), (c) everolimus (1 mg/kg, once daily), or (d) FGF401 (30 mg/kg, twice daily) plus everolimus (1 mg/kg, once daily) for 14 days, as described in Section 4. Throughout the study, all experimental mice exhibited a healthy coat, normal food and water intake, and normal social interactions and activity levels, and there were no signs of aggression among cage mates, indicating minimal toxicity and side effects at the administered doses.

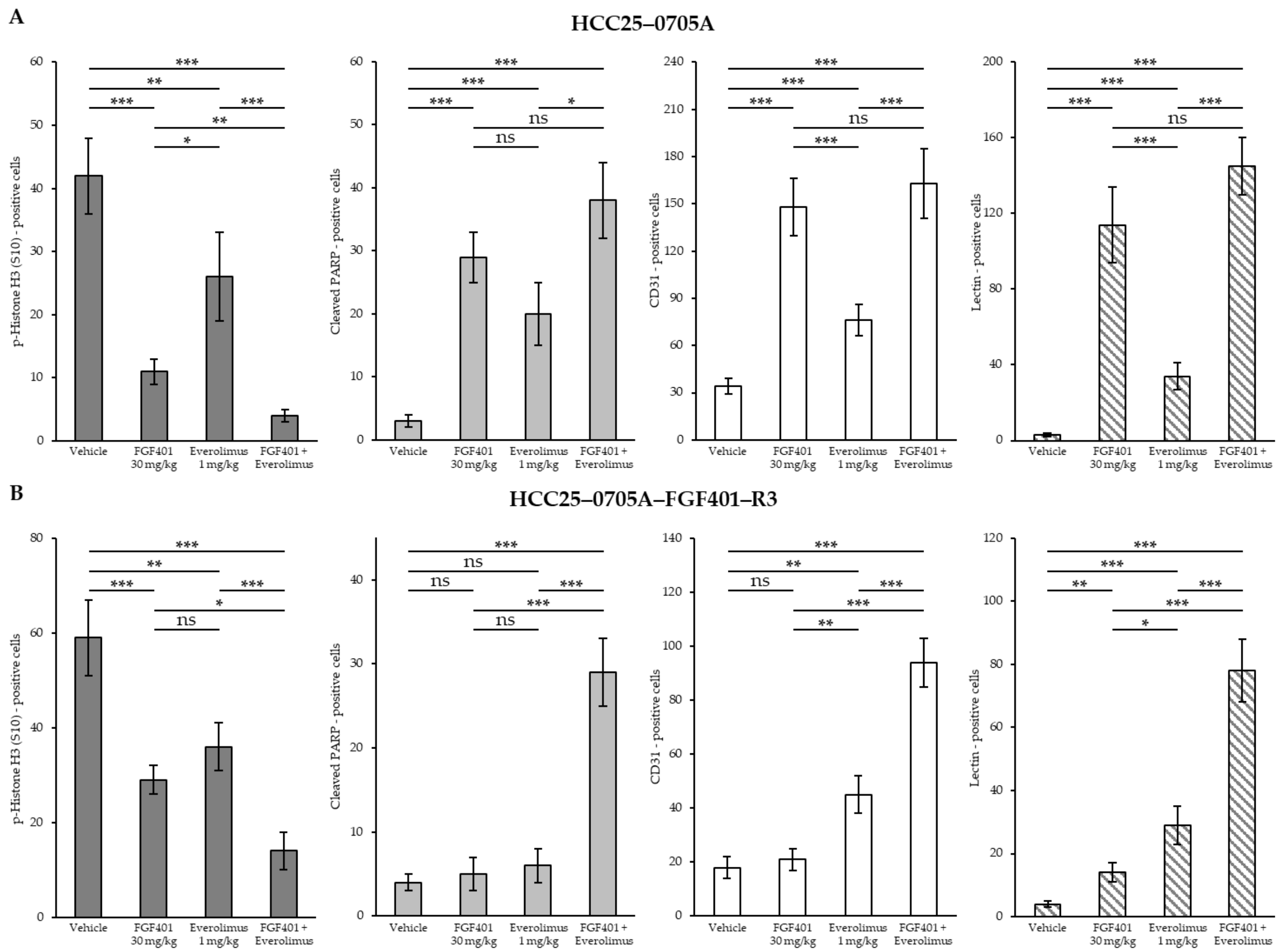

Supplementary Materials Figure S7. Effects of FGF401, everolimus, and FGF401/everolimus on cell proliferation, apoptosis, blood vessel density, and blood vessel normalization in HCC25–0705A and HCC25–0705A-FGF401–R3 PDX models**.** HCC tumors were subcutaneously implanted into SCID mice and treated orally with (a) vehicle (200 µl), (b) FGF401 (30 mg/kg, twice daily), (c) everolimus (1 mg/kg, once daily), or (d) FGF401 (30 mg/kg, twice daily) plus everolimus (1 mg/kg, once daily) as described in Figure 4. Tumor tissues were collected 2 h after the final treatment, fixed in 10% buffered formalin, processed, and paraffin-embedded for IHC, as described in Section 4. Sections (5 µm) were immunostained with p-histone H3 (Ser10), cleaved PARP, CD31, and lectin antibodies to assess cell proliferation, apoptosis, total blood vessels, and blood vessel normalization, respectively. At least 10 randomly selected fields per slide were captured at 100× magnification using an Olympus BX60 microscope (Olympus, Tokyo, Japan). The total number of p-histone H3 (Ser10)-, cleaved PARP-, CD31-, and lectin-positive cells were counted and expressed as mean ± SE. Statistical significance was determined by one-way analysis of variance (ANOVA) followed by Tukey's test (* $p < 0.05$; ** $p < 0.01$; *** $p < 0.001$; ns, no significance).

HCC26–0808B

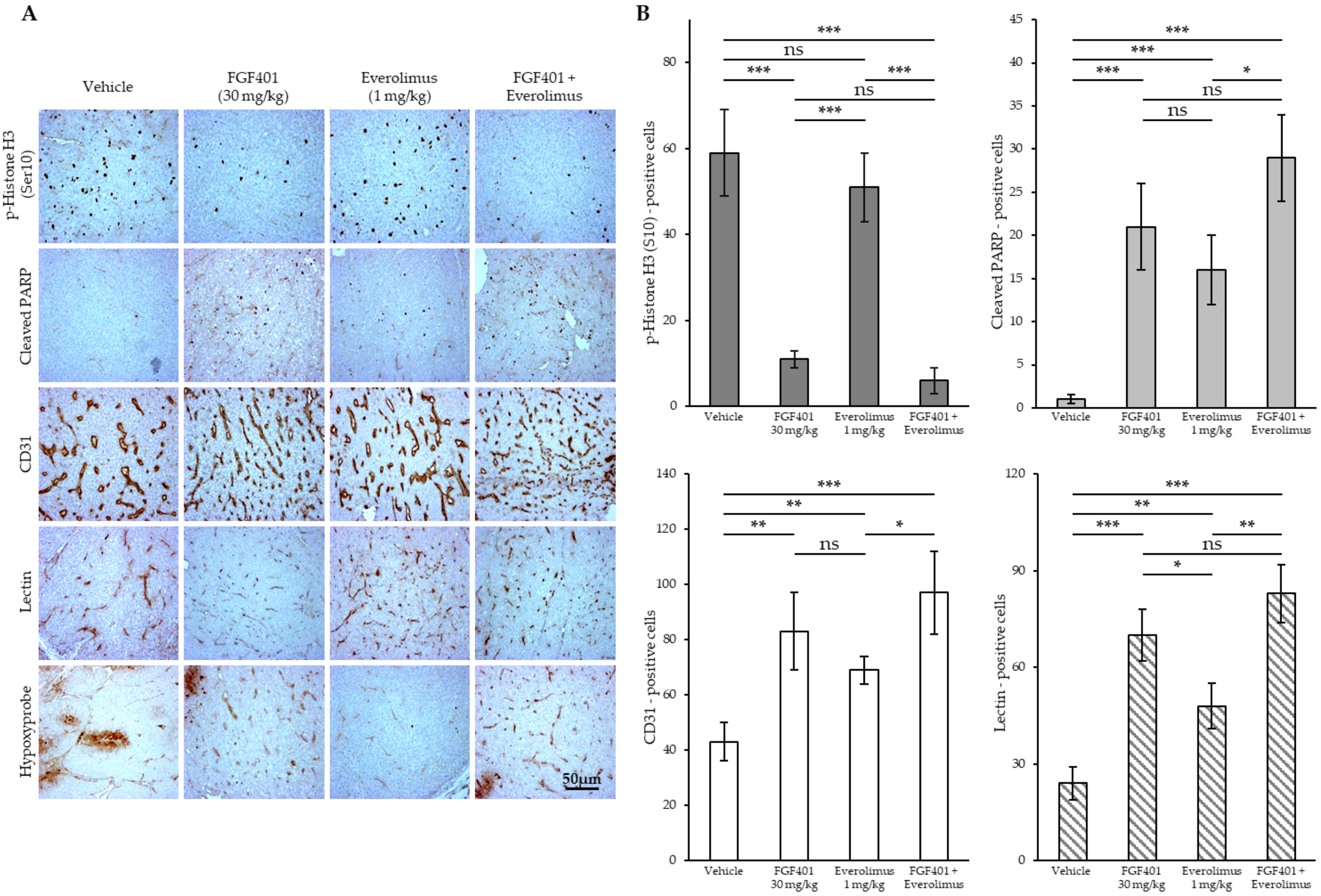

Supplementary Materials Figure S8. Effects of FGF401, everolimus, and FGF401/everolimus on cell proliferation, apoptosis, blood vessel density, blood vessel normalization, and tumor hypoxia in the HCC26–0808B PDX model. HCC tumors were subcutaneously implanted into SCID mice and treated orally with (a) vehicle (200 μl), (b) FGF401 (30 mg/kg, twice daily), (c) everolimus (1 mg/kg, once daily), or (d) FGF401 (30 mg/kg, twice daily) plus everolimus (1 mg/kg, once daily). Tumor tissues were collected 2 h after the final treatment, fixed in 10% buffered formalin, processed, and paraffin-embedded for IHC, as described in Section 4. Sections (5 μm) were immunostained with p-histone H3 (Ser10), cleaved PARP, CD31, lectin, and Hypoxyprobe antibodies to assess cell proliferation, apoptosis, total blood vessels, blood vessel normalization, and hypoxia, respectively. (**A**) Representative tumor sections from vehicle- and drug-treated mice stained for p-histone H3 (Ser10), cleaved PARP, CD31, lectin, and Hypoxyprobe antibodies are shown. Images were captured using an Olympus BX60 microscope (Olympus, Tokyo, Japan). Scale bars = 50 μm. (**B**) Quantification of p-histone H3 (Ser10)-positive cells, cleaved PARP-positive cells, CD31-positive vessels, and lectin-positive vessels. Data are expressed as mean ± SE. Statistical significance was determined by one-way analysis of variance (ANOVA) followed by Tukey's test (* $p < 0.05$; ** $p < 0.01$; *** $p < 0.001$; ns, no significance).

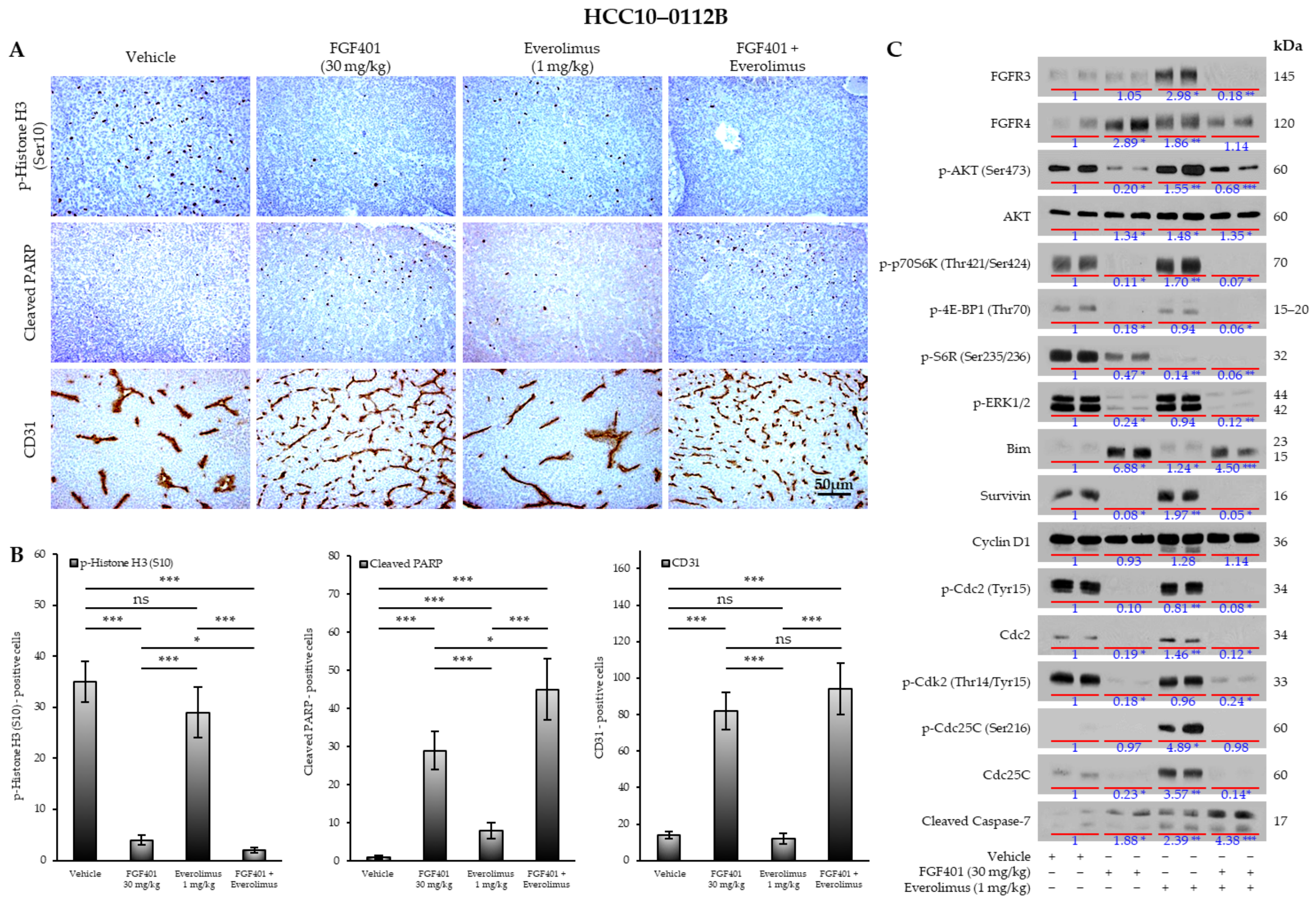

Supplementary Materials Figure S9. Effects of FGF401, everolimus, and FGF401/everolimus on cell proliferation, apoptosis, blood vessel density, and expression of FGFRs and downstream targets in the HCC10–0112B PDX model. HCC tumors were subcutaneously implanted into SCID mice and treated orally with (a) vehicle (200 µl), (b) FGF401 (30 mg/kg, twice daily), (c) everolimus (1 mg/kg, once daily), or (d) FGF401 (30 mg/kg, twice daily) plus everolimus (1 mg/kg, once daily) for 10 days. Tumor tissues were collected 2 h after the final treatment, fixed in 10% buffered formalin, processed, and paraffin-embedded for IHC as described in Section 4. Sections (5 µm) were immunostained with p-histone H3 (Ser10), cleaved PARP, and CD31 antibodies to assess cell proliferation, apoptosis, and total vessel density, respectively. (**A**) Representative tumor sections from vehicle- and drug-treated mice stained for p-histone H3 (Ser10), cleaved PARP, and CD31 antibodies are shown. Images were captured using an Olympus BX60 microscope (Olympus, Tokyo, Japan). Scale bars = 50 µm. (**B**) Quantification of p-histone H3 (Ser10)-positive cells, cleaved PARP-positive cells, and CD31-positive vessels. Data are expressed as mean ± SE. (**C**) Tumor lysates were prepared and subjected to Western blotting and quantitative analysis as described in Section 4. Representative blots probed with the indicated antibodies, the relative expression levels of proteins (expressed as fold changes compared with vehicle-treated samples), and their molecular weights (kDa) are shown. Statistical significance was determined by one-way analysis of variance (ANOVA) followed by Tukey's test (* $p < 0.05$; ** $p < 0.01$; *** $p < 0.001$; ns, no significance.)

HCC29–1104

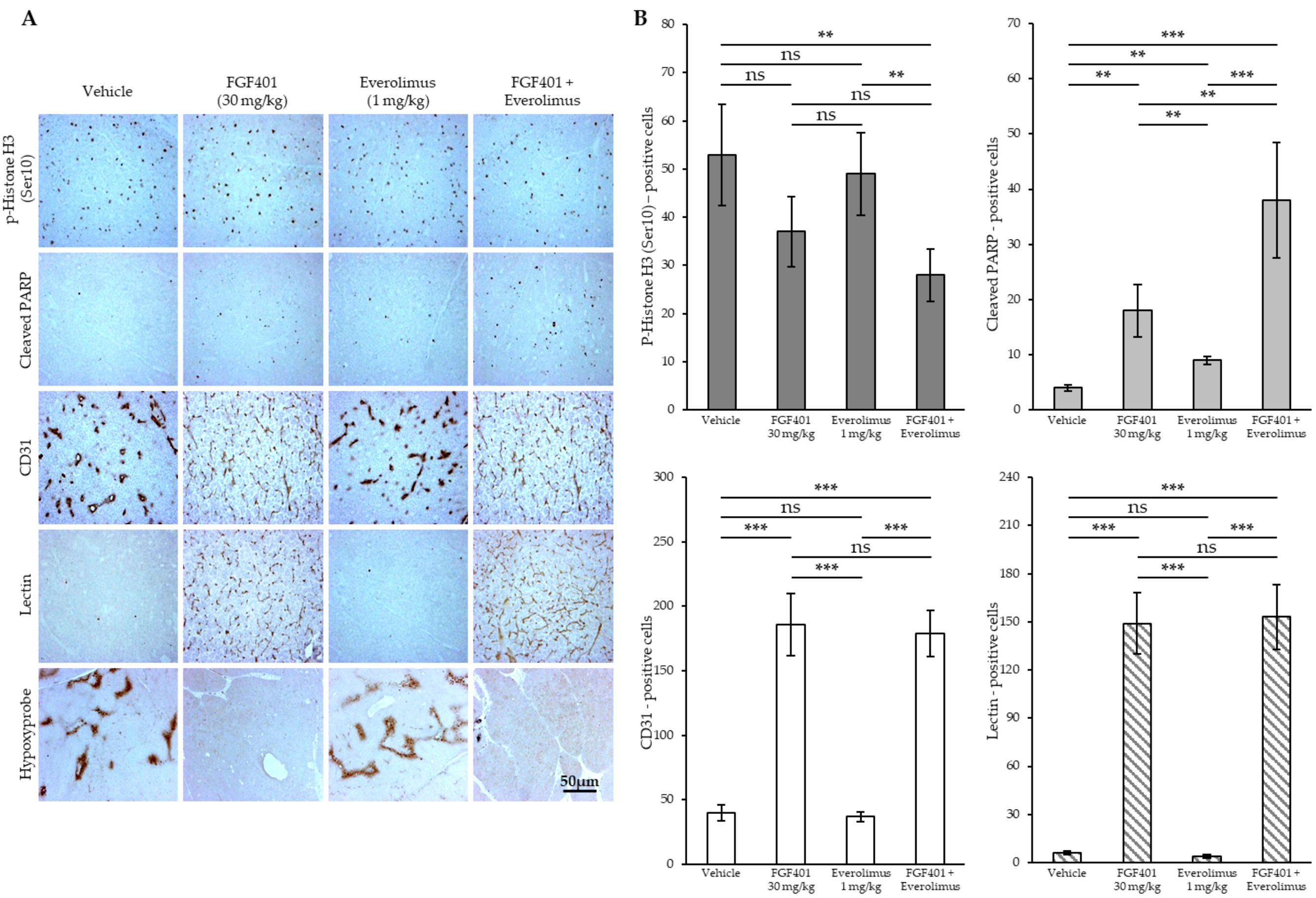

Supplementary Materials Figure S10. Effects of FGF401, everolimus, and FGF401/everolimus on cell proliferation, apoptosis, blood vessel density, blood vessel normalization, and tumor hypoxia in the HCC29–1104 PDX model**.** HCC tumors were subcutaneously implanted into SCID mice and treated orally with (a) vehicle (200 μl), (b) FGF401 (30 mg/kg, twice daily), (c) everolimus (1 mg/kg, once daily), or (d) FGF401 (30 mg/kg, twice daily) plus everolimus (1 mg/kg, once daily) for 9 days. Tumor tissues were collected 2 h after the final treatment, fixed in 10% buffered formalin, processed, and paraffin-embedded for IHC as described in Section 4. Sections (5 μm) were immunostained with p-histone H3 (Ser10), cleaved PARP, CD31, lectin, and Hypoxyprobe antibodies to assess cell proliferation, apoptosis, total blood vessels, and blood vessel normalization, and hypoxia, respectively. (**A**) Representative tumor sections from vehicle- and drug-treated mice stained for p-histone H3 (Ser10), cleaved PARP, CD31, lectin, and Hypoxyprobe antibodies are shown. Images were captured using an Olympus BX60 microscope (Olympus, Tokyo, Japan). Scale bars = 50 μm. (**B**) Quantification of p-histone H3 (Ser10)-positive cells, cleaved PARP-positive cells, CD31-positive vessels, and lectin-positive cells. Data are expressed as mean ± SE. Statistical significance was determined by one-way analysis of variance (ANOVA) followed by Tukey's test (** $p < 0.01$; *** $p < 0.001$; ns, no significance).

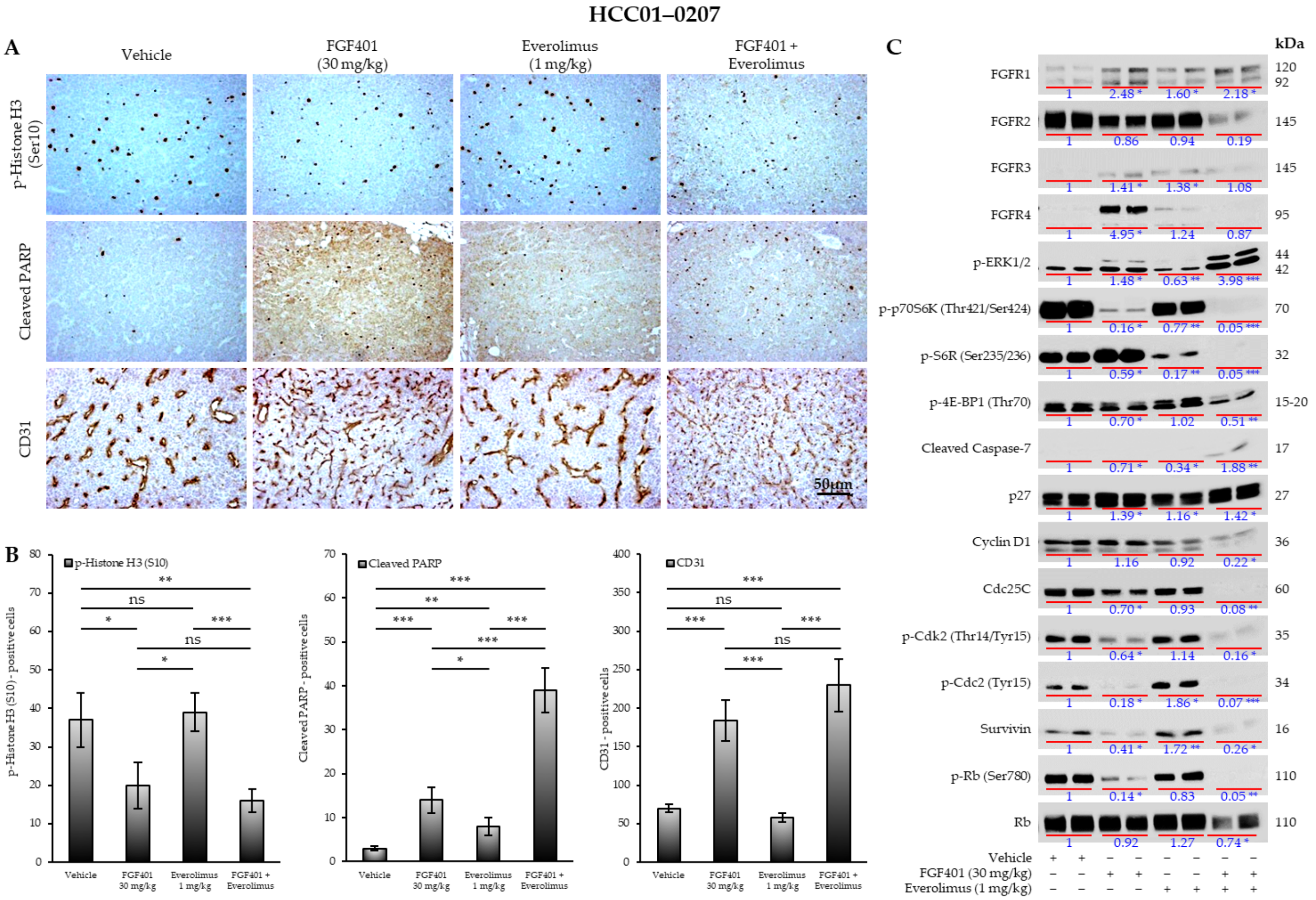

Supplementary Materials Figure S11. Effects of FGF401, everolimus, and FGF401/everolimus on cell proliferation, apoptosis, blood vessel density, and expression of FGFRs and downstream targets in the HCC01–0207 PDX model**.** HCC tumors were subcutaneously implanted into SCID mice and treated orally with (a) vehicle (200 µl), (b) FGF401 (30 mg/kg, twice daily), (c) everolimus (1 mg/kg, once daily), or (d) FGF401 (30 mg/kg, twice daily) plus everolimus (1 mg/kg, once daily) for 10 days. Tumor tissues were collected 2 h after the final treatment, fixed in 10% buffered formalin, processed, and paraffin-embedded for IHC as described in Section 4. Sections (5 µm) were immunostained with p-histone H3 (Ser10), cleaved PARP, and CD31 antibodies to assess cell proliferation, apoptosis, and total vessels density, respectively. (**A**) Representative tumor sections from vehicle- and drug-treated mice stained for p-histone H3 (Ser10), cleaved PARP, and CD31antibodies are shown. Images were captured using an Olympus BX60 microscope (Olympus, Tokyo, Japan). Scale bars = 50 µm. (**B**) Quantification of p-histone H3 (Ser10)-positive cells, cleaved PARP-positive cells, and CD31-positive vessels. Data are expressed as mean ± SE. (**C**) Tumor lysates were prepared and subjected to Western blotting and quantitative analysis as described in Section 4. Representative blots probed with the indicated antibodies, the relative expression levels of protein (expressed as fold changes compared with vehicle-treated samples), and their molecular weights (kDa) are shown. Statistical significance was determined by one-way analysis of variance (ANOVA) followed by Tukey's test (* $p < 0.05$; ** $p < 0.01$; *** $p < 0.001$; ns, no significance.)

Supplementary Table S1. List of primary antibodies used in Western blot analysis.

No.	Antibody	Target Protein	Brand	Cat. No.	Host
1	p-4EBP1 (Thr70)	Phosphorylated eukaryotic translation initiation factor 4E-binding protein 1 at Thr 70	Cell Signaling Technology	#13396	Rabbit
2	AKT	AKT Serine/Threonine Kinase	Cell Signaling Technology	#9272	Rabbit

3	p-AKT (Ser473)	Phosphorylated AKT Serine/Threonine Kinase at Ser 473	Cell Signaling Technology	#9271	Rabbit
4	Bcl-xL	BCL2-like 1	Cell Signaling Technology	#2764	Rabbit
5	Bim	Endogenous levels of total Bim (EL, L and S isoforms) protein	Cell Signaling Technology	#2819	Rabbit
6	Cdc2	Cyclin-Dependent Kinase 1	Santa Cruz Biotechnology	sc-954	Rabbit
7	p-Cdc2 (Tyr15)	Phosphorylated Cell-Dependent Kinase 1 at Tyr 15	Cell Signaling Technology	#9111	Rabbit
8	CDC25C	Cell Division Cycle 25C	Cell Signaling Technology	#4688	Rabbit
9	p-CDC25C (Ser216)	Phosphorylated Cell Division Cycle 25C at Ser 216	Cell Signaling Technology	#4901	Rabbit
10	Cdk2	Cyclin-Dependent Kinase 2	Cell Signaling Technology	#2546	Rabbit
11	p-Cdk2 (Thr14/Tyr15)	Phosphorylated Cyclin-Dependent Kinase 2 at Thr 14/Tyr 15	Santa Cruz Biotechnology	sc-28435-R	Rabbit
12	Cleaved Caspase-3	Activated caspase-3 cleaved at Asp 175	Cell Signaling Technology	#9661	Rabbit
13	Cleaved Caspase-7 (Asp198)	Endogenous levels of the large fragment of caspase-7 resulting from cleavage at aspartic acid 198	Cell Signaling Technology	#9491	Rabbit
14	Cyclin D1	Cyclin D1	Cell Signaling Technology	#2978	Rabbit
15	eIF4E	Eukaryotic translation initiation factor 4E	Cell Signaling Technology	#9742	Rabbit
16	p-eIF4E (Ser209)	Phosphorylated Eukaryotic translation initiation factor 4E at Ser 209	Cell Signaling Technology	#9741	Rabbit

17	ERK1/2	p44 and p42 MAP Kinase (Erk1 and Erk2)	Santa Cruz Biotechnology	sc-94	Rabbit
18	p-ERK1/2	Phosphorylated p44 and p42 MAP Kinase (Erk1 and Erk2) at Thr202 and Tyr204	Cell Signaling Technology	#4370	Rabbit
19	FGFR1	Endogenous levels of total FGF receptor 1 protein	Cell Signaling Technology	#9740	Rabbit
20	FGFR2	Endogenous levels of total FGF receptor 2 protein	Cell Signaling Technology	#11835	Rabbit
21	FGFR3	Endogenous levels of FGF Receptor 3 protein	Cell Signaling Technology	#4574	Rabbit
22	FGFR4	Endogenous levels of total FGF receptor 4 protein.	Cell Signaling Technology	#8562	Rabbit
23	P27	Cip/Kip family of cyclin-dependent kinase inhibitor 1B	Cell Signaling Technology	#3686	Rabbit
24	p-p70S6k (Thr389)	Phosphorylated ribosomal protein S6 kinase B1 at Thr 389	Cell Signaling Technology	#9205	Rabbit
25	p-p70S6k (Thr421/Ser424)	Phosphorylated ribosomal protein S6 kinase B1 at Thr 421/Ser 424	Cell Signaling Technology	#9204	Rabbit
26	p-PI3K p85 (Tyr458)/p55 (Tyr199)	Endogenous levels of p85/p55 protein only when phosphorylated at Tyr467/Tyr199	Cell Signaling Technology	#17366	Rabbit
27	Rb	Retinoblastoma (Rb) transcriptional corepressor 1	Cell Signaling Technology	#9313	Rabbit
28	p-Rb (Ser780)	Phosphorylated Rb at Ser 780	Cell Signaling Technology	#8180	Rabbit
29	S6R	Endogenous levels of total S6 ribosomal protein independent of phosphorylation	Cell Signaling Technology	#2217	Rabbit
30	p-S6R (Ser235/236)	Phosphorylated ribosomal protein S6 at Ser 235/236	Cell Signaling Technology	#4858	Rabbit

31	Survivin	Survivin	Cell Signaling Technology	#2808	Rabbit
32	α-Tubulin	Endogenous levels of total α-tubulin protein	Cell Signaling Technology	#3873	Mouse
